# Uneven Use of Remote Work to Prevent the Spread of COVID-19 in South Korea's Stratified Labor Market

**DOI:** 10.3389/fpubh.2021.726885

**Published:** 2021-10-13

**Authors:** Saejung Park, Sanghee Lee, Joonmo Cho

**Affiliations:** ^1^Human Resource Development Center Researcher, Department of Economics, Sungkyunkwan University, Seoul, South Korea; ^2^Department of Consilience, Korea Polytechnic University, Siheung, South Korea; ^3^Department of Economics, Sungkyunkwan University, Seoul, South Korea

**Keywords:** COVID-19, remote work, dual labor market, polarization, collective bargaining, revision of employment rules unfavorably to workers

## Abstract

**Background:** This research analyzed whether South Korean companies adopted remote work during the COVID-19 pandemic by focusing on the dual labor market structure comprising of primary sector (large corporations) and secondary sector [small and medium enterprises (SMEs)]. Companies in the dual labor market were classified based on firm size.

**Methods:** We used August supplementary data from the Economically Active Population Survey covering 2017–2020 provided by Statistics Korea. In this empirical study, a Linear Probability Model was used to analyze the probability that employees would work for companies that introduced remote work since COVID-19 depending on the size of the company.

**Results:** This study showed three main results. First, unlike other flexible work systems, the use of remote work has increased rapidly since COVID-19. Second, the larger the size of the company, the higher the probability that employees would work for companies that introduced remote work after COVID-19. Third, according to the analysis by industry, the difference in remote work utilization between large corporations and SMEs was relatively small because of a similar working method in manufacturing.

**Conclusion:** Results of this study suggested that polarization within the dual labor market structure also spilled over to adoption of remote work, which was initially introduced to prevent the spread of the pandemic. This study examined the system and factors of labor-management relations contributing to such polarization and presented policy directions for the current labor market structure.

## Introduction

A lockdown as one of the most stringent measures to combat the spread of a virus has resulted in the halt of production and services. Businesses worldwide have increased the use of remote work to continue corporate activities during this period. In Denmark, Netherlands, and Sweden, employees working from home have increased after the pandemic-induced lockdown (1).

In Republic of Korea (henceforth, simply South Korea), although remote work had already been adopted before the pandemic, only 3% of companies implemented it in 2016 (2). A Ministry of Employment and Labor survey in 2019 regarding the intention of firms to introduce remote work showed that only 4% of respondents intended to adopt remote work, indicating that the reluctance of South Korean businesses to implement this policy (3). However, remote work has emerged as a prominent temporary measure to maintain a certain level of production and services amidst setbacks and as a measure to prevent the spread of COVID-19 in South Korea (4).

Although the use of remote work is expected to continue even in the post-pandemic era, the extent of its adoption is likely to vary depending on the labor environment of each country and characteristics of its economic entities. This difference is likely to lead to variations in the effect of remote work on the labor market (5). Some scholars have argued that polarization, one of the major issues faced by the South Korean labor market, could be a likely cause affecting the adoption of remote work by firms during the COVID-19.

Therefore, the impact of a polarized labor market on remote work implemented during the pandemic needs to be analyzed so that measures could be devised to address this issue. To this end, aims of this study were: (1) to check whether the use of remote work had increased since the outbreak of COVID-19; (2) to determine whether there was a difference in the use of remote work after the outbreak of COVID-19, focusing on the size of the company; and (3) to determine whether there was a difference in the use of remote work after the outbreak of COVID-19, focusing on dividing the industry into manufacturing and service. A primary sector (large corporations) was defined when the number of employees was more than 300. A secondary sector (SMEs) was defined if the number of employees was <300. Based on research results, the use of remote work amidst the pandemic was confirmed, with a focus on the dual labor market structure. Also, manufacturing and service industries differ in their working methods. Therefore, results were analyzed by taking the type of industry into consideration.

Based on results of an empirical analysis showing that polarization within a dual labor market structure could also be spilled over into whether companies adopted remote work, which was initially introduced to prevent the spread of the pandemic, this study examined the system and factors of labor-management relations contributing to such polarization and presented policy directions for the current labor market structure. Previous literature, the dual labor market in South Korea, the trend of COVID-19 in South Korea and use of remote work, data and analysis methods, results, and the legal process for the establishment of remote work in primary and secondary sectors are described in order.

## Literature on Remote Work During Covid-19 and the Labor Market Polarization

### COVID-19 Outbreak and Deepening of the Labor Market Polarization

COVID-19 has severely and adversely affected economies of each country, industries, and the daily lives of citizens. Although impact of the pandemic on the labor market varied from country to country, it accelerated the polarization that existed even before its outbreak. Numerous recent studies have predicted the possibility that the pandemic may further intensify polarization in the local labor market.

The OECD announced that COVID-19 would especially impact low-wage and unstable jobs, and workers from these types of jobs would be more seriously affected by the social distancing rule and lockdown measures in the service sectors such as restaurants and hotels (6). Furthermore, the OECD maintained that due to the coronavirus, self-employed, temporary workers, and part-time laborers were significantly exposed to risk of unemployment and income loss, and that the lockdown measures taken by the European members of the OECD could adversely affect nearly 40% of the jobs in such vulnerable sectors (7).

The World Bank has also stressed the importance of support in hiring and maintaining the productivity of vulnerable, informal economy workers and small firms to cope with the negative effect of COVID-19 (8). Additionally, the ILO also highlighted negative impact of the pandemic on SMEs, and small business owners, the self-employed, informal economy workers, temporary workers, and new types of workers working in the gig economy. COVID-19 is expected to further aggravate labor poverty and inequality because its negative effect is more damaging to small business owners and workers who were already vulnerable (9). An IMF Working Paper also warned that the COVID-19 outbreak could deepen inequity in Asia, especially related to gender-based income inequality and economic imbalance between cities and rural areas (10).

Empirical studies reporting about the pandemic-induced polarization in the local labor market also presented similar predictions. A US-based study on the effect of COVID-19 on job markets argued that the reduction in hiring due to the pandemic was the most prominent in low-income communities and areas with a wide income gap. The study also found that a fall in hiring was the most severe in industries with a high unionization rate and in local service sectors such as education, public health, retail, and construction (11).

Some studies reported that the coronavirus pandemic particularly adversely affected the female workers. They found that in the United States, married women were more likely to have experienced reduced work hours or job loss due to COVID-19, suggesting its long-term effect on female employment and the deepening of gender inequality (12). In addition, other studies found that whereas cyclical economic downturns had a more significant impact on male jobs, social distancing rules amidst COVID-19 had greater impact on the employment of female workers than males (13).

### Working From Home Amidst COVID-19 Pandemic and Labor Market Polarization

Working from home is necessary for reducing economic loss while maintaining economic activity during the pandemic, and can also potentially improve other social and economic indicators such as productivity, employee welfare, and reduce local income inequality (14). In most countries, work from home has been largely induced by the coronavirus pandemic. Nevertheless, although the OECD has cited the advantages of working from home as a response to the pandemic, in reality, its use has been confined to only a limited number of workers. In fact, in the UK and Europe, prior to COVID-19, remote work was only allowed to high-paying employees such as managers, professionals, public administrators, and other senior business staff (15). In contrast, after the pandemic, low-income workers are more likely to lose their jobs because they are ill prepared for remote work and are pessimistic about continuing earning income through remote work, whereas people in high-income positions are 50% more likely to work remotely (16). Furthermore, the COVID-19 crisis is prompting employers to extend remote working opportunities where possible, leading to greater investment in remote work infrastructures, which could bring some long-term benefits. However, these measures would not help frontline workers who cannot work remotely and are more exposed to infection (17). An International Monetary Fund Working Paper reported that after the COVID-19 outbreak, hiring was most severely hit in sectors where remote work is not possible, such as service sector jobs in hospitality and tourism industries. In addition, workers from industries where remote work is not affordable are more likely to earn lesser average income than those in other industries. Thus, overall, the pandemic would exacerbate income inequality in sectors where remote work is not possible (18).

Empirical studies have found that the COVID-19 pandemic will further deteriorate the labor market inequality between workers who can work remotely and those who cannot. Studies that analyzed the practice of remote work in the UK, the US, and Germany after the pandemic found that in all three countries, workers who can work from home during the pandemic are far less likely to lose their jobs, whereas workers exposed to the risk of infection are more likely to become unemployed. Moreover, in the US and the UK, workers who work remotely for fewer hours are more likely to experience a decrease in income (19).

In Germany, a study that assessed employment inequality during the lockdown from the first wave of the pandemic found that while low-income workers seriously suffered from unemployment, employees with superior qualifications could afford to work remotely. Employees who continue to work from home are much less concerned about their job security than those who cannot their change work hours or workplaces. Additionally the infection risk only increased for individuals who began working on-site after being laid-off (20). In addition, some researchers analyzed the impact of increased remote work opportunities on the labor market in Italy during the pandemic, which has the lowest rate of remote work among the European countries. They found that the rise of remote work benefited males, the elderly, and workers with good education and high income, which could most likely reinforce wage inequality that had existed prior to the pandemic (21).

The probability of safe working environments through measures such as remote work stems from two factors. The first factor is technology intensity. The second factor is the work conditions before the pandemic. For instance, those who earned high income prior to the pandemic and could afford to work even during lockdowns are more likely to work safely at home. Hence, remote work indicates the possibility of an increase in income polarization (22).

Working from home during the pandemic is slated to help maintain economic activities, reduce economic loss, as well as potentially boost or improve social and economic indicators such as productivity and employee welfare, while reducing local inequality. Developing countries that have an inadequate digital infrastructure must focus on introducing or modifying policies, laws, and regulations in many sectors to reap the benefits of remote work, including digitalization and other related practices (14).

## The Impact of Dual Labor Market Structure on Remote Work During Covid-19 in South Korea

### Trend of COVID-19 Spread in South Korea and the Use of Remote Work

**Figure 1:**
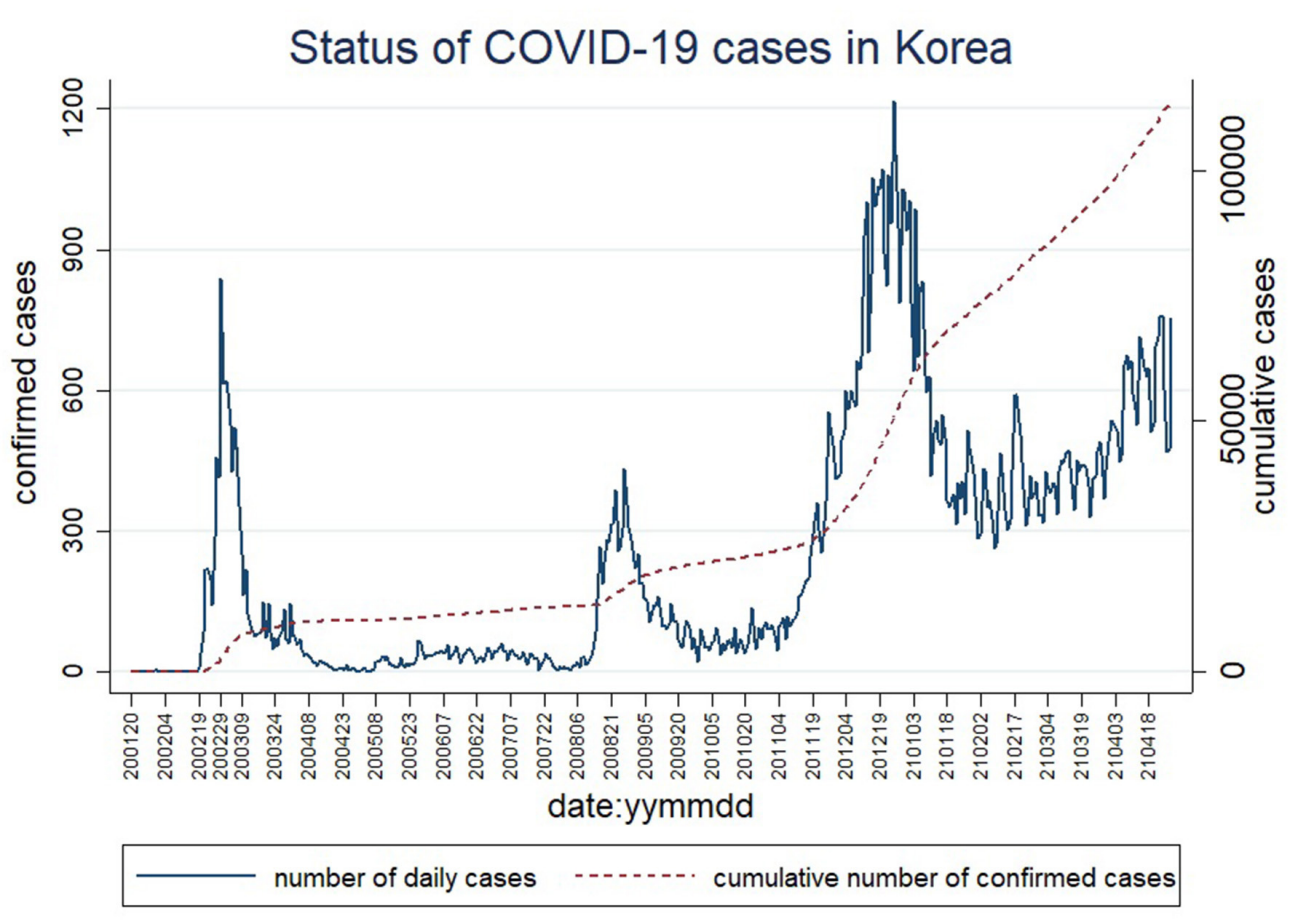
Daily trend and cumulative trend of COVID-19 confirmed cases in South Korea. Both the daily trend and the cumulative trend of COVID-19 confirmed cases use the number of people as a unit. The first two digits of the six digits of the date indicate the year 2020 or 2021. The middle two digits represent January through December. The last two digits represent the date from the 1st to the 31st.

Figure 1 shows the spread of COVID-19 cases in Korea. Since the first confirmed case of COVID-19 in Korea, the government announces the status of confirmed cases every day (http://ncov.mohw.go.kr/). Daily confirmed cases were collected directly. Figure 1 was prepared using such data. The X axis represents the timeline from January 20, 2020, when the first COVID-19 case occurred in the country, to February 2020. The Y axis on the left indicates daily cases, while the y axis on the right refers to cumulative cases. The solid line shows the number of cases per day and the dashed line indicates the cumulative number of cases. Figure 1 confirmed that South Korea had three massive outbreaks during this period. The first wave happened in February and March 2020, with viral spread due to large-scale religious gatherings in Daegu and Gyeongbuk areas attended by coronavirus-infected individuals who had previously visited China. During the first wave, people were afraid of being infected with Covid-19. Therefore, the South Korean government took strong measures such as a ban on movement between regions, social distancing, and remote work to nip the rapid spread of the contagion. At that time, companies began to introduce remote work. Owing to these efforts, by April 2020, cases dropped sharply. The world praised South Korea for its efforts to contain the pandemic. Nevertheless, the sudden adoption of the remote work system was ill-equipped to sustainably tackle the economic crisis at home and abroad. Therefore, many businesses eventually began to revert to an offline mode of work (23, 24).

The second wave occurred after a rally in downtown Seoul held around the National Liberation Day on August 15, 2020. Seoul and metropolitan areas surrounding the capital city had the highest spike in cases. Consequently, the government took a stern measure by banning meetings involving five or more persons in the Seoul metropolitan area and reducing service hours of restaurants and supermarkets. The government decided to reinstate remote work to prevent the spread of the infection (25, 26).

The third wave of the pandemic began due to a sharp rise in cases in November 2020. Daily number of cases surpassed the record number of 1,200. This accelerating infection rate revived citizens fear infection again. By February 2021, cases had dropped to around 400, easing an upward trend. However, daily cases have failed to fall further. Realizing that the pandemic would not end in the near future, South Korean businesses have started building remote work infrastructure (27) for the future, while relying on offline work when the pandemic has slowed down and implementing remote work when cases have surged.

### Impact of Dual Labor Market Structure on Labor Market Polarization in South Korea

Labor market polarization in South Korea was already present prior to the coronavirus pandemic. In general, labor market polarization in South Korea is synonymous with its dual labor market structure (28), which is comprised of the primary sector, including large corporations, and regular employees with labor unions, and the secondary sector representing SMEs and non-regular workers without the support of union.

Table 1 shows the number of workers, average monthly wages, and their work years in primary and secondary sectors of the labor market. Table 1 used the same data used in the main analysis, the August 2019 supplementary survey of the Economically Active Population Survey (EAPS) of South Korea Statistics (https://mdis.kostat.go.kr/). A detailed description of the data is provided in the data section. As of 2019, primary sector and secondary sector represented 4.4% (859,237 persons) and 27.9% (4,991,345 persons) of total wage earners in South Korea, respectively. Both sectors had a significant gap in their working conditions. The average monthly income of workers in the primary sector was 2.57 times that of the secondary sector workers. The primary sector workers worked about six times the number of years worked by secondary sector workers. The large wage gap between the two sectors can be attributed to difference in earnings to the pay-out between large corporations and SMEs as well as differences in their wage practices. That is, in Korea, large businesses will increase wages of employees in proportion to the number of years worked, following a seniority-based wage system.

**Table 1:** Dual labor market structure of Korea.

**Category**	**Primary labor market**	**Secondary labor market**
(1) Share of workers (*N*, %)	859,237, 4.4	4,991,345, 27.9
(2) Wage (KRW)	4,128,000	1,603,900
(3) tenure (Years)	14.47	3.21

*(1) Primary labor market refers to regular workers who joined a labor union while working for large corporations*.

*(2) Secondary labor market refers to non-regular workers who did not join the labor union while attending small and medium-sized enterprises (SMEs)*.

*(3) In the table, row (1) refers to share of total number of salaried workers. Row (2) indicates average monthly wage of workers in each market. Row (3) represents average tenure of workers in each market*.

In Korea, educated male workers are likely to occupy the primary sector (29). Regular positions mostly involve white-collar jobs employing disproportionate number of male workers with degree of Bachelor. Additionally, the primary sector offers a highly automated and digitalized working environment. Hence, the workers in the primary sector can easily adapt to remote work during the pandemic. Moreover, 62.9% of the large companies in the primary sector have labor unions; therefore, workers in the primary sector are free from the risk of losing jobs and hardly experience significant variation in wages and salaries (30, 31).

Meanwhile, the secondary sector comprises of non-regular workers in SMEs, of which only 10% are unionized. That is, companies in the secondary sector have fewer earnings to pay for wages and because their employees are not unionized, they are less likely to protect their jobs or build the infrastructure required to achieve digitalization.

### Polarization in the Use of Remote Work Among South Korean Firms

In South Korea, remote work saw a sharp increase as a temporary measure in several companies to maintain business activities that were suspended due to COVID-19. However, the use of remote work in South Korea is highly limited by its dual labor market structure, which further intensified during the coronavirus pandemic, showing that dual labor market structure and remote work influence each other reciprocally. Remote work is mostly prevalent among large corporations (32), whereas nearly half the SME employees reported to work on-site amidst the polarization between the two types of companies (33). In addition, public institutions reported double the rate of remote work use compared to SMEs (34).

Most of the workers in the secondary labor market are least likely to have an option to work remotely. Additionally, companies in the secondary labor market have insufficient financial resources to pay out wages compared to those in the primary sector, and thus cannot afford to continue paying wages to employees working from home. Consequently, due to its inability to offer remote work opportunities, workers in the secondary sector have a higher chance of unemployment during the pandemic than the primary sector.

## Methods

### Data

To analyze characteristics of South Korean workers who worked from home before and after the outbreak of the pandemic, we required pre- and post-outbreak remote work data. The August supplementary survey by the Economically Active Population Survey (EAPS) of South Korea Statistics (https://mdis.kostat.go.kr/) provided us with such data. South Korea Statistics provided the original data of the EAPS and the august supplementary survey by the EAPS. These data were approved by Statistics Korea to be used by all persons who had applied through MDIS, the website of Statistics Korea. This research analyzed raw EAPS data from MDIS.

The EAPS focuses on the labor supply data collected through household visits each month, which is used as a base data to investigate the monthly employment and unemployment rates. In addition to the monthly EAPS, the August supplementary survey divides workers into salaried and non-salaried workers depending on labor type of the respondents, and collects additional information about labor quality through data on labor contracts, labor hours, and employment insurance by labor type. Thus, since 2001, the EAPS August supplementary survey has been providing detailed information on approximately 35,000 households by economic activity and labor type, as of August. This survey also offers data on the use of flexible work arrangements by salaried workers, including remote work. Thus, it is useful to analyze the trends and characteristics of employees working remotely before and after the outbreak of the pandemic.

This study analyzed the characteristics of the workers who worked remotely before and after the outbreak of the coronavirus pandemic and measured the effect of the dual labor market structure on remote work. This study considered employees who reported receiving flexible work opportunities from their employers a week before the survey, and identified the use of remote work as respondents (samples) answering “work from home” or “remote work” in response to the question “Which type of flexible work system do you use?.” The samples did not include salaried workers who are engaged in agriculture, forestry, and fishery; those engaged in domestic activities; and instances of self-consumption and production activities that are not classified into any specific category. In addition, to compare the pre- and post-outbreak data, the analysis period covered every August from 2017 to 2020, where August 2020 belongs to the period after the coronavirus outbreak. Among the samples satisfying these conditions, we eliminated those containing missing values in the explanatory variables and finally included 100,136 samples in our analysis.

### Methodology

This study used the linear probability model (LPM) to analyze the use of remote work among salaried workers. The LPM can be used when the dependent variable is not continuous and discrete. The dependent variable, which is the focus of this study, is a binary variable indicating whether the company where employee works used remote work. During the analysis period, the use of remote work by salaried workers was indicated as 1 and non-use of remote work was labeled as 0. When a dependent variable was a binary variable, it was analyzed mainly using a logit or probit model. When there was interaction term in non-linear model, Ai and Norton (2003) highlighted the issue of interaction effects in non-linear models (35). To resolve this, this paper used LPM to address this problem.


$$\begin{array}{l} y_{it}=\{\begin{array}{c} 1,\;if\;z_{it}>0\\ 0,\;if\;z_{it}\leq 0 \end{array} \end{array}$$

(1) $$\begin{array}{l} where\;z_{it}=\;\beta_{0}+\;\overset{K}{\underset{k=1}{\sum }}\beta_{k}x_{kit}+u_{it} \end{array}$$

In formula (1), the subscript *i* is an individual, and *t* is time. *x*_*it*_ indicates the explanatory variables related to the personal characteristics and job characteristics of individuals *i* during *t* time. The explanatory variables included age, residence area, gender, education, marital status, position at workplace, occupation, the number of employees (firm size), and the year dummy variable.

In formula (1), *z*_*it*_ represents the sum of a linear combination of the constant and explanatory variables and the error term. Considering *E*(*u*_*it*_) = 0 to provide an unbiased estimate, *E*(*y*_*it*_|*X*_*it*_), which is a conditional expectation of *y*_*it*_ *given X*_*it*_, is a conditional probability of *y*_*it*_ = 1 and expressed below as formula (2) (36).


(2) $$\begin{array}{l} E\left[y_{it}|X_{it}\right]=\mathrm{Pr}\left(y_{it}|X_{it}\right)=\beta_{0}+\overset{K}{\underset{k=1}{\sum }}\beta_{k}x_{kit} \end{array}$$

Furthermore, to determine the impact of the firm size on the use of remote work after the outbreak of COVID-19, total number of employees and the year 2020 were added as interaction terms. In formula (3), the expected values were added to both sides of the regression equation to interpret interaction effect.


(3) $$\begin{array}{l} E\left[y_{it}|X_{it}\right]=\mathrm{Pr}\left(y_{it}|X_{it}\right)=\beta_{0}\\ \;+\sum_{j}\beta_{j}\cdot 1\left\{total\;number\;of\;employees_{it}=j\right\}\\ \;+\sum_{k=2018}^{2020}\beta_{k}\cdot 1\{t=k\}\\ \;+(\sum_{j}\sum_{k=2018}^{2020}\delta_{jk}\cdot 1\left\{total\;number\;of\;employees_{it}=j\right\}\\ \;\times 1\left\{t=k\right\})+X_{it}^{\prime }\alpha \end{array}$$

In formula (3), *i* is the number of employees of a firm where an individual *i* works. The number of employees is a categorical variable with four groups: 1–4 persons (base), 5–29 persons, 30–299 persons, and 300 persons or more. κ is a categorical variable that divides the analysis period into four: 2017 (base), 2018, 2019, and 2020. *X*_*it*_ is a variable that represents the personal and job characteristics of an individual (*i*), which also represent the explanatory variables used in formula (2).

## Empirical Analysis

### Basic Statistical Analysis

**Figure 2:**
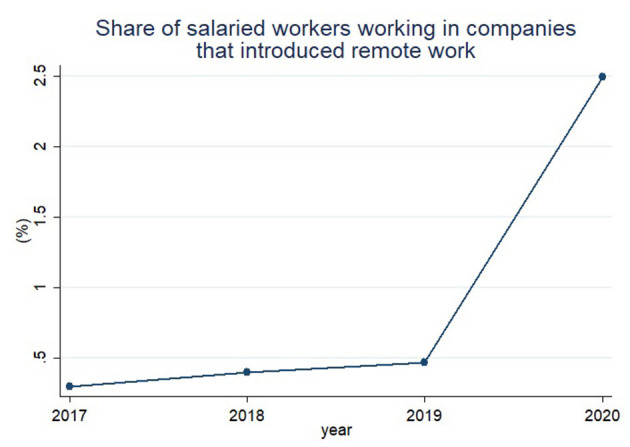
It shows share of salaried workers working in companies that introduced remote work.

Figure 2 shows the proportion of salaried workers who worked remotely out of the total number of salaried workers, based on the EAPS August supplementary survey. The share of remote workers steadily surged from 0.30% in 2017 to 0.40% in 2018, and 0.47% in 2019. In 2020, after the outbreak of COVID-19 pandemic, the share of workers attending companies that implemented remote work soared by five times to 2.49% from 2019.

**Figure 3:**
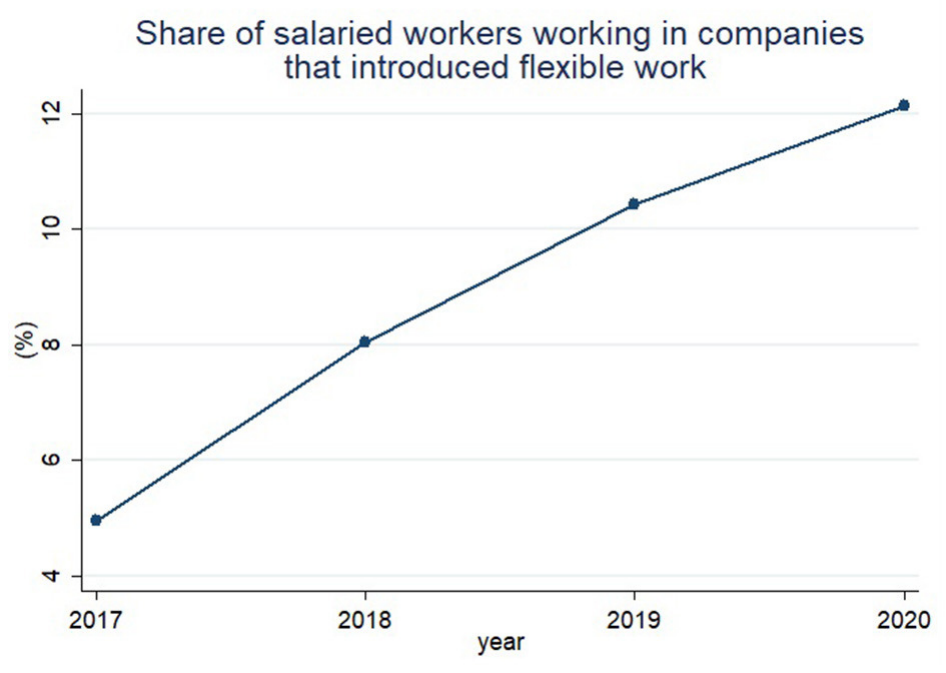
It shows share of salaried workers working in companies that introduced flexible work arrangements except for remote work.

Figure 3 presents the proportion of salaried workers who have worked in companies with flexible work arrangements except for remote work. The share of workers using flexible work systems steadily increased from 4.7% in 2017 to 12.0% in 2020. That is, unlike Figures 2, 3 shows a steady increase in the proportion of salaried workers using flexible work arrangements regardless of COVID-19. Figure 3 shows that remote work among flexible work arrangements is heavily affected by COVID-19.

Table 2 presents the annual statistics of salaried workers working for companies that implemented remote work. Specifically, it shows that among the salaried workers employed in companies with the remote work option, the share workers residing in *dong* (urban areas) exceeded that of salaried workers living in *eup/myeon* (rural areas). In 2020, regardless of the region, the share of salaried workers attending companies with remote work rose sharply; however, the share of 2020 relative to 2019 surged by about six times in *dong* (urban) areas, far outranking the increase in *eup/myeon* areas. There was no significant difference between male and female workers working in companies with the remote work option. Additionally, the higher the education level, the higher the share of workers with companies allowing remote work. In particular, the share of workers with the degree of Masters in remote-based roles rose to 6.9% in 2020. Meanwhile, on an average, during the survey period, only 0.5% of the workers who graduated from a technical college or lower were employed by companies that offered a remote work option. However, irrespective of the education levels, the share of workers with remote-based jobs rose by seven times during 2019 to 2020, showing the most prominent increase for all educational levels. In 2017, the share of employees working remotely did not vary significantly with marital status: unmarried persons 0.1%, married persons 0.3%, and the divorced/widowed 0.2%; however, in 2020, the share of both married and unmarried persons rose to 2.2% while that of divorced/widowed workers climbed to 0.5%, representing a substantial change.

**Table 2:** Annual statistics on workers employed in companies offering remote work.

**Main category**	**Middle category**	**Variables**	**2017**	**2018**	**2019**	**2020**	**Mean**
Personal characteristics	Region	Cities (%)	0.3	0.4	0.4	2.3	0.8
		Rural (%)	0.1	0.1	0.2	0.8	0.3
	Gender	Male (%)	0.2	0.3	0.4	2	0.7
		Female (%)	0.3	0.5	0.4	2	0.8
	Education	High school or lower (%)	0.1	0.1	0.1	0.4	0.2
		Technical college or lower (%)	0.2	0.3	0.2	1.4	0.5
		University or lower (%)	0.4	0.6	0.7	3.6	1.4
		Master's degree or higher (%)	0.6	1.2	1.2	6.9	2.5
	Marital	Unmarried (%)	0.1	0.2	0.4	2.2	0.7
	status	Married (%)	0.3	0.4	0.5	2.2	0.9
		Divorced /widowed (%)	0.2	0.2	0.1	0.5	0.2
Job characteristics	Occupation	Manager (%)	0.4	0.7	1	4.5	1.7
		Clerical workers (%)	0.4	0.6	0.6	3.8	1.3
		Service workers (%)	0	0.1	0.1	0.3	0.1
		Sales workers (%)	0.5	0.5	0.5	2	0.9
		Technicians (%)	0	0.1	0	0.3	0.1
		Workers of simple labor (%)	0	0	0	0.1	0
	Industry	Manufacturing (%)	0.1	0.2	0.4	1.2	0.4
		Construction, other manufacturing (%)	0.1	0.2	0.2	0.4	0.2
		Wholesale and retail, food, accommodation (%)	0.2	0.3	0.3	1.1	0.4
		Transport, communication (%)	0.4	0	0	1.4	0.5
		Finance, insurance, real estate (%)	0.5	0.4	0.4	3.5	1.2
		Public social and personal services (%)	0.1	0.4	0.4	3.3	1
		Education, healthcare, social service, art (%)s	0.2	0.4	0.3	2	0.8
		Others (%)	0.5	0.7	0.9	4.1	1.5
	Number of employees	1–4 (%)	0.1	0.1	0.2	0.3	0.2
		5–29 (%)	0.3	0.3	0.4	1	0.5
		30–299 (%)	0.2	0.5	0.5	2.9	1
		300 or more (%)	0.4	0.6	0.6	6	1.9
Total			0.2	0.3	0.4	2	0.7

*Total in the last row represents employees' rate employed in companies offering remote work in each year*.

Regarding job characteristics, managers constituted the most significant figure (1.7%), followed by clerical workers (1.3%) and sales workers (0.9%). The share of managers who attended companies offering remote work has steadily risen since 2017. The share of clerical workers in 2018 and 2019 showed a change from 0.4 and 0.6%, indicating less steady growth than the managers. However, the ratio showed a 6-fold increase from 2019 to 2020. In terms of industry type, the share of remote workers was higher in finance, insurance, real estate, public social service, personal service, and others. Sectors such as finance, insurance, real estate, transportation/communication, public social service, personal service, and others showed a sharp increase in the share of workers working remotely from 2019 to 2020. The size of companies was found to be positively related to the share of workers engaged in remote work. The share for companies with 1–4 employees was 0.2%, 5–29 employees was 0.5%, 30–299 employees was 1.0%, and those with 300 plus employees was 1.9%. Large companies employing more than 300 workers saw the share of workers engaging in remote work soaring by ten times from 0.6% in 2019 to 6% in 2020.

**Figure 4:**
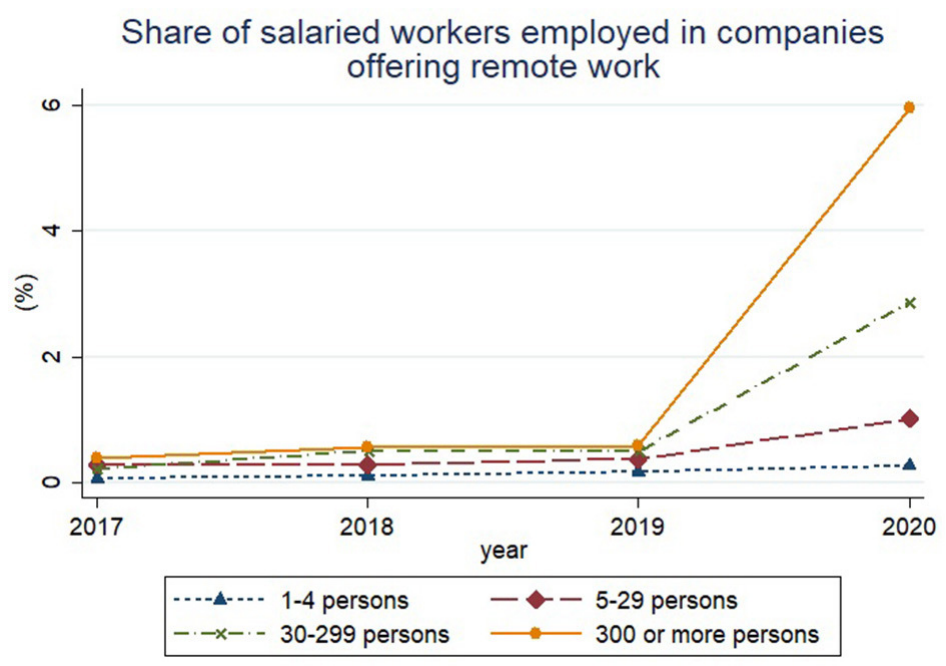
It indicates share of salaried workers employed in companies offering remote work divided by company size. The company size was divided into four groups based on the number of employees. The four groups consist of small businesses with 1–4 employees, small enterprises with 5–29 employees, medium-sized enterprises with 30–299 employees, and large corporations with more than 300 employees.

Figure 4 shows the trends for the share of salaried workers working in companies implementing remote work by firm size and year, based on data from Table 2. The X axis indicates the year, and the Y axis indicates the share of salaried workers working in companies with remote work opportunity. The dotted line with a triangle marker indicates the share of salaried workers working in companies with 1–4 employees. The dashed line with a diamond marker represents firms with 5–29 employees, the dash-dotted line with an X marker indicates firms with 30–299 employees, and the solid line with a circle marker shows the share of workers attending large companies with over 300 employees. Regardless of the number of employees, we found that the share of workers working from home steadily rose from 2017 to 2020. Notably the share in 2020 climbed sharply in proportion to the firm size.

## Results

Table 3 presents the result of the analysis from considering “whether the company where employee works used remote work” as the dependent variable. The results were derived by using the Linear Probability Model, which included the variables likely to affect whether the companies introduced remote work. Results of Table 3 are similar to the results of the basic statistics in Table 2. Specifically, regarding the personal characteristics variables, employees with a degree of Master are more likely to work for companies with remote work system by 1.09 percentage points.

**Table 3:** Results of analysis of the dependent variable.

**Main category**	**Middle category**	**Variables**	**Status of introduction of remote work**	
			**Coef**.	**Robust** **Std. Err**.
Personal characteristics	Age	Age	−0.0001^**^	0.000
	Residence area	Base: Cities		
		Rural	−0.0012^**^	0.001
	Gender	Base: Male		
		Female	0.0029^***^	0.001
	Education	Base: High school or lower		
		Technical college	−0.001	0.001
		University	0.0040^***^	0.001
		Master's degree or higher	0.0109^***^	0.002
	Marital status	Base: Unmarried		
		Married	0.0033^***^	0.001
		Divorced/widowed	0.0027^***^	0.001
Job characteristics	Employment status	Base: Regular positions		
		Temporary, day laborers	−0.0021^***^	0.001
	Occupation	Base: Service workers		
		Managers and professionals	0.0097^***^	0.001
		Clerical workers	0.0061^***^	0.001
		Sales workers	0.0054^***^	0.001
		Technicians	0	0.001
		Workers engaged in simple labor	−0.0012^*^	0.001
	Industry	Base: Manufacturing		
		Construction and other manufacturing	0.0012	0.001
		Wholesale and retail trade, food, accommodation	0.0034^***^	0.001
		Transportation/communication	0.0026^**^	0.001
		Finance, insurance, real estate	0.0042^***^	0.002
		Public social service, personal service	0.0040^***^	0.001
		Education, healthcare, social service, arts	−0.0021^**^	0.001
		Others	0.0091^***^	0.001
	Number of employees	**Base: 1–4 persons**		
		5–29 persons	0.0031^***^	0.001
		30–299 persons	0.0070^***^	0.001
		300 or more persons	0.0128^***^	0.001
	Log (average wage of recent three months)		0.0020^***^	0.001
	Working hours		−0.0001^***^	0
Year	Year dummy	**Base: 2017**		
		2018	0.0008	0
		2019	0.0013^**^	0.001
		2020	0.0178^***^	0.001
Constant			−0.0145^***^	−0.003
Sample Size			100,136	
R-squared			0.019	

*(1) Dependent variable in this result is whether the company where employee works used remote work*.

*(2) Statistically significant at significance level of*

*** *1%*,

** *5%*,

* *10%*.

Regarding job characteristics-related variables, the number of employees within a firm is particularly relevant. Employees in large companies with over 300 employees are 1.28 percentage points more likely to work remotely than employees working in small companies with 1–4 employees. For the year variable, salaried workers are increasingly more likely to work for companies offering remote work opportunities as the year approached 2020, compared with 2017. Additionally, the coefficient of the correlation was prominent in year 2019 and 2020 and the probability of workers engaged in remote work was higher by 1.78 percentage points in 2020 than in 2017.

Table 4 lists the results of the analysis of whether adoption of remote work by companies was influenced by firm size after the outbreak of the pandemic. That is, by checking the interaction term between the number of employees in a firm and the COVID-19 period variable, we measured the variation in the probability of introduction of remote work of the company after the COVID-19 depending on its number of employees. As in Table 3, our analysis considered the dependent variable as a dummy variable with value 1 if companies with salaried workers provide remote work opportunities, and value 0 if not. The explanatory variables for personal characteristics and job characteristics of the employee remained the same as in Table 3. We then measured the interaction effect of the number of employees in a firm and year variables using the year dummies.

**Table 4:** Interaction term analysis using the number of employees in a firm and year variables.

**Main category**	**Middle category**	**Variables**	**Status of introduction of remote work**	
			**Coef**.	**Robust** **Std. Err**.
Job characteristics	Status of employment	Base: Regulated employees		
		Temporary, day laborers	−0.0016^**^	0.001
	Number of employees	**Base: 1–4**		
		5–29	0.0017^**^	0.001
		30–299	−0.0004	0.001
		300 or more	−0.0016	0.001
Period	Year dummy	**Base: 2017**		
		2018	0.0001	0.001
		2019	0.0005	0.001
		2020	0.0015^*^	0.001
Interaction term	Number of employees X Year	**Base: 1–4 employees X 2017**		
		(1) X 2018	−0.0003	0.001
		(1) X 2019	0.0001	0.001
		(1) X 2020	0.0056^***^	0.001
		(2) X 2018	0.0021^*^	0.001
		(2) X 2019	0.0019	0.001
		(2) X 2020	0.0251^***^	0.002
		(3) X 2018	0.0013	0.002
		(3) X 2019	0.0008	0.002
		(3) X 2020	0.0540^***^	0.004
Control variables	Personal characteristics		O	
	Occupation		O	
	Industry		O	
	Log (average wage in the past three months)		−0.0016^**^	−0.001
	Actual hours of employment in a major job		−0.0002^***^	0
Sample size			100,136	
R-squared			0.026	

*(1) Dependent variable in this result is whether the company where employee works used remote work*.

*(2) In the table, column 3 refers to interaction term for company size and year. (1) In column 3 represents there are 5–29 employees in the company. (2) Indicates there are 30–299 employees in the company. (3) Indicates the company has more than 300 employees*.

*(3) Statistically significant at significance level of*

*** *1%*,

** *5%*,

* *10%*.

Our analysis found that, considering firms with 1–4 employees for the year 2017 as the base, in 2020, employees from companies with 5–29 employees were 0.56 percentage points more likely to work from home, and employees from companies with 30–299 employees were 2.51 percentage points more likely to work remotely compared to the base case. For companies with 300 employees, the probability of employees working from home in 2020 rose by 5.40 percentage points from the base case. Similar to Tables 3, 4 shows that workers attending companies with a size larger than 1–4 employees after the outbreak of COVID-19 are proportionately more likely to work from home.

Tables 5, 6 show results of analysis by dividing the sample into manufacturing and service industries. The manufacturing industry consisted of manufacturing, construction, and other manufacturing sectors. The service industry consisted of including wholesale and retail, food and accommodation, etc. The reason for analyzing samples by industry was because the working method differed depending on the industry. There were also differences in the utilization of remote work. In addition, even within the same industry, there were differences in working methods depending on the size of the company, which might affect the utilization of remote work. Table 5 shows whether there is a difference in the utilization of remote work after the outbreak of COVID-19 depending on the size of the company for workers engaged in the manufacturing industry. Table 6 analyzes workers in the service industry.

**Table 5:** Interaction term analysis for the manufacturing industry.

**Main category**	**Middle category**	**Variables**	**Status of introduction of remote work**	
			**Coef**.	**Robust** **Std. Err**.
Interaction term	Number of employees X Year	**Base: 1–4 employees X 2017**		
		(1) X 2018	0.0025	(0.002)
		(1) X 2019	0.0029	(0.002)
		(1) X 2020	0.0040	(0.003)
		(2) X 2018	0.0021	(0.002)
		(2) X 2019	0.0020	(0.002)
		(2) X 2020	0.0060^**^	(0.003)
		(3) X 2018	0.0005	(0.002)
		(3) X 2019	0.0044	(0.003)
		(3) X 2020	0.0232^***^	(0.005)
Control variables	Personal characteristics		O	
	Work characteristics		O	
	Year dummy		O	
Sample size			28,870	
R-squared			0.013	

*1) Dependent variable in this result is whether the company where employee works used remote work*.

*2) It analyzed only workers in the manufacturing industry*.

*3) In the table, column 3 refers to interaction term for company size and year. (1) In column 3 represents there are 5-29 employees in the company. (2) Indicates there are 30–299 employees in the company. (3) Indicates the company has more than 300 employees*.

*4) Statistically significant at significance level of*

*** *1%*,

** 5%, *10%.

**Table 6:** Interaction term analysis for the service industry.

**Main category**	**Middle category**	**Variables**	**Status of introduction of remote work**	
			**Coef**.	**Robust Std. Err**.
Interaction term	Number of employees X Year	**Base: 1-4 employees X 2017**		
		(1) X 2018	−0.0011	(0.001)
		(1) X 2019	−0.0005	(0.001)
		(1) X 2020	0.0066^***^	(0.002)
		(2) X 2018	0.0028^*^	(0.002)
		(2) X 2019	0.0027^*^	(0.002)
		(2) X 2020	0.0352^***^	(0.003)
		(3) X 2018	0.0027	(0.003)
		(3) X 2019	−0.0004	(0.003)
		(3) X 2020	0.0721^***^	(0.006)
Control variables	Personal characteristics		O	
	Work characteristics		O	
	Year dummy		O	
Sample Size			71,266	
R-squared			0.033	

*1) Dependent variable in this result is whether the company where employee works used remote work*.

*2) It analyzed only workers in the service industry*.

*3) In the table, column 3 refers to interaction term for company size and year. (1) In column 3 represents there are 5-29 employees in the company. (2) Indicates there are 30–299 employees in the company. (3) Indicates the company has more than 300 employees*.

*4) Statistically significant at significance level of*

*** 1%, **5%,

* *10%*.

Manufacturing is main industry in South Korea. It has a similar production method regardless of the size of the company. It is difficult to introduce remote work except for some jobs in large corporations. In addition, SMEs are unlikely to use remote work because of limitations of their working methods and the vulnerable digital environment. According to results shown in Table 5, only workers working for large corporations with more than 300 employees increased the probability of working in companies using remote work by 2.3 percentage point since the COVID-19 outbreak. The coefficient value was less than half that of Table 4. Table 5 shows that the difference in remote work utilization between large corporations and SMEs is relatively small because of a similar working method.

Unlike manufacturing, the service industry differs greatly in terms of working method between large corporations and SMEs. Large corporations operate mainly on-site services through outsourcing except for internal main management. Therefore, it is difficult for SMEs to use remote work compared to large corporations. Results of Table 6 showed that employees, regardless of the size of the company, increased the probability of working in companies using remote work. In addition, the larger the company, the more likely the remote work would be used by employees. However, due to differences in working methods, the probability of working for companies through remote work increased to 7.21 percentage point after the COVID-19 outbreak, widening the gap between employees of large corporations and employees of SMEs.

### Robustness Check

Samples were reconstructed to test the robustness of the results analyzed in Table 4. If workers changed jobs after the COVID-19 outbreak or had a special employment, it might affect the probability of working for a company that introduced remote work. Therefore, when the survey year was August 2020, workers who worked for the company for <8 months were excluded from the sample.

Results are as follows. Employees for companies with 5–29 employees were 0.60 percentage points more likely to work from home and employees for companies with 30–299 employees were 2.54 percentage points more likely to work remotely compared to the base case. For companies with 300 employees, the probability of employees working from home in 2020 rose by 5.54 percentage points from the base case. In other words, Table 7 shows results of analysis after excluding samples that might have transferred to companies that adopted telecommuting during COVID-19. This confirms that results in Table 4 are robust.

**Table 7:** Robustness check of Table 4's results.

**Main category**	**Middle category**	**Variables**	**Status of introduction of remote work**	
			**Coef**.	**Robust Std. Err**.
Interaction term	Number of employees X Year	**Base: 1–4 employees X 2017**		
		(1) X 2018	0.0004	(0.001)
		(1) X 2019	0.0006	(0.001)
		(1) X 2020	0.0060^***^	(0.002)
		(2) X 2018	0.0016	(0.001)
		(2) X 2019	0.0017	(0.001)
		(2) X 2020	0.0254^***^	(0.002)
		(3) X 2018	0.0013	(0.002)
		(3) X 2019	0.0013	(0.002)
		(3) X 2020	0.0554^***^	(0.005)
Control variables	Personal characteristics		O	
	Work characteristics		O	
	Year dummy		O	
Sample Size			95,189	
R-squared			0.027	

*(1) In the table, column 3 refers to interaction term for company size and year. (1) In column 3 represents there are 5–29 employees in the company. (2) Indicates there are 30–299 employees in the company. (3) Indicates the company has more than 300 employees*.

*(2) Statistically significant at significance level of*

*** 1%, **5%, *10%.

## Implications of Increased Use of Remote Work in South Korea in the Post-Pandemic Era

### Procedure of Labor Relation Laws Governing Remote Work in South Korea

As discussed earlier, the remote work system in South Korea was introduced mostly as a temporary measure after the outbreak of the pandemic to stem the spread of the COVID-19, and was adopted without any adjustment process. This conclusion stems from the variation in the level of remote work depending on the fluctuations of COVID-19 caseloads in South Korea. The decision to establish a remote work system is not expected to receive any opposition from the employees if it is implemented while keeping intact the current task assessment and remuneration schemes.

However, the first challenge of allowing remote work is to devise methods to measures the work attitude and task performance of remote workers. Because of the difficulties in controlling their work status on a real-time basis while they work from home, companies would need to modify their performance assessment and compensation system. This would hardly be supported by employees because unlike a temporary use of remote work during the pandemic, a long-term system would involve a strict assessment of employees working status and performance, as well as wage reduction.

Introducing a remote work system in South Korea involves two legal procedures. The first is to revise the collective agreement between companies and employees. Whether employees agree to the introduction of remote work proposed by employers would be determined by collective bargaining. Trade unions and employers may decide whether to introduce remote work after an adjustment of performance assessment and compensation system.

The second procedure for introducing remote work involves an amendment to the employment rules. Employment rules are rules about working conditions unilaterally prescribed by employers in order to systematically and consistently control the working conditions of employees. According to the current laws, the employment rules cannot breach the provisions of a collective (37); however, unless a collective agreement specifically bans the use of remote work, the employment rules can be revised to introduce remote work. Meanwhile, in case the amendment of the employment rules is favorable to the employees, obtaining the consent of the trade unions or the majority of the employees is not necessary. However, if the employment rules are amended in a way that disadvantages the interests of the workers, the amendment must obtain the consent of the trade union or the majority of workers (38). For instance, if while establishing a remote work system the employers wish to impose a strict performance evaluation or wage adjustments that might deteriorate the current working conditions of the workers, the amendment would require the consent of the majority of workers.

Furthermore, recently the Supreme Court of South Korea established a precedent that even after obtaining a majority consent, an amendment to the employment rule that puts workers at a disadvantage would have no effect for a certain worker unless a labor contract with the said individual worker is revised accordingly (Supreme Court, November 14, 2019, Sentence 2018 da 200709) (39). This implies that even if the introduction of remote work was decided through the difficult process of amending the employment rules, the use of remote work cannot be finalized in case the amendment requires an additional procedure of revising the labor contract with individual workers. This suggests that the current legal environment makes it difficult to introduce remote work at workplaces.

### Rigidity in Collective Bargaining in the Primary Sector

In South Korea, the primary sector labor market saw an increase in the adoption of remote work after the outbreak of the COVID-19; however, depending on the fluctuation of caseloads, the use of remote work and the return to office-based work occurred without an adjustment in the working conditions. That is, due to the unique circumstances during the pandemic, companies implemented remote work without applying special procedures such as obtaining a majority consent of the workers. However, going forward, in the post-pandemic era, the continued use of remote work in the primary sector may not be easily accommodated by labor organizations if the existing performance assessment and remuneration schemes are modified.

In South Korea, a trade union representing two-thirds or more workers of a particular workplace, can sign a union shop clause that would allow the trade union to force an organization to meet its demands (Trade Union Act §94-1) (38). For example, trade unions of large companies who have signed the union shop provision can make all eligible employees to become members of the trade unions. Hence, the high prevalence of trade unions within large primary sector companies in South Korea implies that companies cannot implement a remote work policy without a collective agreement that such a policy. Even when a collective agreement does not have a provision that prohibits the introduction of remote work, an amendment to the employment rules for the insertion of a remote work system requires the consent of the trade union or a majority votes of the workers. Therefore, with good cooperation between labor organizations and the management regarding corporate competitiveness and productivity is essential for the insertion of provisions on the use of remote work into collective agreement or employment rules. However, the negotiation culture between labor organizations and the management in South Korea has not been cooperative, so much so that the national competitiveness in terms of the labor-management relations is a major source of concern. In fact, among 140 countries, South Korea ranked 135th in 2016 and 2017, 124th in 2018, and 130th in 2019 in the labor-management relations assessment confirmed by the World Economic Forum (40).

Nevertheless, remote work is likely to continue in the post-pandemic era in a way that benefits both employer and employees. Accordingly, companies that anticipate the use of remote work need to establish improved systems and conditions allowing employees to choose a remote work. This is because remote work requires companies to modify their existing business performance and compensation framework, which is currently suitable only for the existing mode of work.

### Rigidity in Revising Unfavorable Employment Rules for Secondary Sector Workers

The secondary sector faced considerable obstacles for implementing remote work during the pandemic. SMEs with non-regular workers in the secondary sector reduced workforce instead of offering remote work opportunities due to a lack of financial resources to pay wages to the workers. While the primary sector driven by large corporations, responded to the pandemic by suspending hiring and reducing costs, the secondary sector mostly comprised of SMEs, had to lay off even their skilled workers (41). However, after the pandemic even the SMEs would need to consider introducing remote work.

In case the secondary sector intends introduce remote work, similar to the primary sector, the workers are unlikely to accept the policy easily if it involves modifications to the existing task performance evaluation and compensation methods. As the secondary sector pays workers a smaller compensation amount, it cannot afford to adjust performance assessment and remuneration schemes implementing remote work. Nevertheless, the use of remote work in the primary sector is likely to influence its adoption in the secondary sector.

In the secondary labor market, only 12.3% of middle market enterprises with 122–299 employees and 2.7% of medium-sized companies with 30–99 employees have trade unions (30). Moreover, most of the trade unions in the secondary sector are so small that they are poorly equipped to use their collective bargaining power to have their demand accepted through labor strikes and other means. Therefore, the secondary sector companies are more likely to consider introducing remote work through revision of the employment rules instead of collective bargaining. Even so, the introduction of remote work by amending the employment rules requires the consent of the majority of employees, which could pose a challenge. As mentioned earlier, even with the consent of the majority of the employees, if a concerned employee requires a revision of a labor contract, companies must make such a revision. Therefore, overall, the introduction of remote work in the secondary labor market in South Korea is not easy under the current legal system.

## Conclusion

This study empirically analyzed whether companies use remote work after the outbreak of COVID-19, focusing firm size, using the August supplementary survey of the EAPS released by Statistics Korea. Focusing on determining whether the gap in the use of remote work by firm size narrowed in an effort to combat the coronavirus, we found that the probability of large corporations implementing remote work after the outbreak of COVID-19 in 2020 surged more rapidly than small companies, thus widening the gap in the labor market.

Although this research analyzed the use of remote work in connection with the dual labor market, this research had several limitations. The first limitation was that the recent situation could not be included in the analysis due to data limitations. Although the EAPS provides monthly data published by Statistics Korea, the main variable used in this study, whether remote work was used or not, was included in the August Supplementary Survey of the EAPS, which was surveyed every August. The most recent data were the August 2020 data. The recent introduction of vaccines and the spread of mutated viruses were not considered in this analysis.

The second limitation was that we did not fully control the endogeneity problem. Companies that introduced remote work were large, had the ability to pay, and prepared to measure workload and performance online. Employees who worked for these companies might mainly have outstanding competencies in addition to their academic background. In this research, there was a limitation in controlling the competency of workers because panel analysis such as fixed effect could not be applied. Follow-up studies are needed to address these limitations.

Given dual labor market structure in South Korea, companies require customized support to establish a remote work system after the pandemic. The government of South Korea has recently been supporting SMEs to establish remote work infrastructure (42). These efforts may be considered as a policy reflecting the dual labor market structure. Additionally, during the pandemic, the South Korean government designated workers engaging coronavirus prevention efforts, with services employees working on-site, including parcel delivery, frontline workers protecting the safety of ordinary citizens and the socially vulnerable sections, and care-based workers acting as “essential workers.” The South Korean government also implemented policies ensuring the safety and social protection for these people (43). Such policy is similar to protective measures for essential workers in the UK (44) and protection of essential workers and the HEROES Act in the United States (45, 46).

The secondary sector, which faces greater difficulties in implementing remote work, requires rapid, targeted, and intensive support. Without an appropriate support, SMEs in the secondary sector cannot overcome the obstacle of insufficient financial resources, which can hinder their survival and lead to massive unemployment, resulting in a sharp increase in the cost of unemployment benefits. In a country like South Korea, where there is a clear distinction of labor market between primary and secondary sectors, a timely support needs to be provided to eligible targets, without which the disparity in the dual labor market structure will further intensify.

## Data Availability Statement

Publicly available datasets were analyzed in this study. The raw data can be found here: https://mdis.kostat.go.kr/.

## Ethics Statement

Ethical review and approval was not required for the study on human participants in accordance with the local legislation and institutional requirements. Written informed consent for participation was not required for this study in accordance with the national legislation and the institutional requirements.

## Author Contributions

SP: collected data, analyzed the result, wrote and edited initial draft, and revised the draft. SL: provided conceptualization direction and wrote initial draft. JC: provided conceptualization direction, design of collection tools, policy dimensions, and supervision. All authors read and approved the final manuscript.

## Funding

This paper was supported by SKKU Excellence in Research Award Research Fund, Sungkyunkwan University, 2020.

## Conflict of Interest

The authors declare that the research was conducted in the absence of any commercial or financial relationships that could be construed as a potential conflict of interest.

## Publisher's Note

All claims expressed in this article are solely those of the authors and do not necessarily represent those of their affiliated organizations, or those of the publisher, the editors and the reviewers. Any product that may be evaluated in this article, or claim that may be made by its manufacturer, is not guaranteed or endorsed by the publisher.

## Abbreviations

EAPS: Economically Active Population Survey
ILO: International Labor Organization
IMF: International Monetary Fund
LPM: Linear Probability Model
MDIS: Micro Data Integrated Service
OECD: Organization for Economic Cooperation and Development
SMEs: small and medium enterprises
UK: United Kingdom
US: United States.
